# Facts and Mysteries of Spiritism

**Published:** 1886-03

**Authors:** 


### Book Reviews.

Facts and Mysteries of Spiritism. *By* Joseph Hartman. *12 mo, cloth, pp. 378. Philadelphia:* T. W. Hartley & Co. *Chicago:* Jansen, McClurg & Co. 1885.

The verdict of common sense would naturally send the author to an insane asylum; but in that light, Swedenborg, and even St. John, would have been considered insane, as far as their hallucinations or visions are concerned. If Mr. Hartman is insane, we must confess that he is a most intelligent lunatic.

This book is a graphic account of the obsession of the author by evil spirits for a space of time extending over seven years. The terrible effects of these hallucinations could not have been better described by any one than by the sufferer himself. The book will furnish valuable material for the study of insanity, inasmuch as it contains eleven cases of obsession besides that of the author. That such cases are often sent to insane asylums is not surprising, as that is about as good a place as any in which they could be properly taken care of; yet, from the frequency of milder forms of hallucination, in people of perfectly sound mind, a greater discrimination ought to be exercised in the examination of the insane than is done nowadays. This duty ought to be intrusted to physicians possessed of more than plain common sense. This book is well written, and nicely printed. Its theories will play havoc in the spiritualists' camp, for the author, from his familiarity with evil spirits, has ceased to believe that good spirits can manifest themselves to the living. It is to be hoped that the majority of readers are proof against the contagiousness of such hallucinations, which would carry us back to the dark ages when our ancestors walked on four. But Mr. Hartman deserves our sympathy for his peculiar belief, for, hallucinations being mostly reversals to ancestral ideas, and our ancestors having sprung from the quadruped family, one would naturally expect that the revelations of spiritualism would turn out to be nothing but the remembrances of beastly memories.

